# Activin levels correlate with lymphocytic infiltration in epithelial ovarian cancer

**DOI:** 10.1002/cam4.7368

**Published:** 2024-09-09

**Authors:** Elizabeth T. Evans, Emily F. Page, Alex Seok Choi, Zainab Shonibare, Andrea G. Kahn, Rebecca C. Arend, Karthikeyan Mythreye

**Affiliations:** ^1^ Division of Gynecologic Oncology, Department of Obstetrics and Gynecology, Heersink School of Medicine University of Alabama School of Medicine Birmingham Alabama USA; ^2^ Department of Pathology University of Alabama at Birmingham Heersink School of Medicine Birmingham Alabama USA

**Keywords:** activin, metastasis, ovarian cancer, tumor immune infiltration

## Abstract

**Objective:**

The TGF‐β superfamily member activin, a dimer of the gene products of *INHBA* and/or *INHBB,* has been implicated in immune cell maturation and recruitment, but its immune impact within epithelial ovarian cancer (EOC) is not well characterized. We sought to explore differences in activin (*INHBA/* Inhibin‐βA and *INHBB/* Inhibin‐βB) between malignant and ovarian tissues at the RNA and protein level and assess the relationship between activin and immune cells in EOC.

**Methods:**

Publicly available RNA sequencing data were accessed from GEO (#GSE143897) with normalization and quantification performed via DESeq2. Immune gene expression profile was further explored within the TCGA‐OV cohort derived from The Cancer Genome Atlas (TCGA). Immunohistochemical analysis was performed to evaluate activin A and T‐cell markers CD8 and FoxP3 at the protein level. ELISA to activin‐A was used to assess levels in the ascites of advanced EOC patients. Kaplan–Meier curves were generated to visualize survival outcomes.

**Results:**

Gene expression levels of components of the activin signaling pathway were elevated within EOC when compared to a benign cohort, with differences in activin type I/II receptor gene profiles identified. Additionally, *INHBA* gene expression was linked to lymphocytic immune markers in EOC samples. Immunohistochemistry analysis revealed a positive correlation of CD8 and FOXP3 staining with activin A at the protein level in both primary and metastatic epithelial ovarian cancer samples. Furthermore, Activin‐A (inhibin‐βA) is significantly elevated in EOC patient ascites.

**Conclusion:**

*INHBA* expression is elevated within EOC, correlating with worse survival, with activin protein levels correlating with specific immune infiltration. Our findings suggest that activin‐A may play a role in suppressing anti‐tumor immunity in EOC, highlighting its potential as a therapeutic target.

## 1 INTRODUCTION

Ovarian cancer remains the eighth most common cause of cancer‐related deaths worldwide and has the highest mortality rate of the gynecologic malignancies. Within the United States, an estimated 19,880 women were diagnosed with ovarian cancer in 2022, with 12,810 women succumbing to the disease that year.^1^ The majority of epithelial ovarian cancers (EOC) are diagnosed at advanced stages, and unfortunately, both rate of recurrence and chemoresistance are high. A better understanding of the biology that underlies ovarian cancer development and disease progression is needed to help identify therapeutic targets for future intervention.

Emerging evidence supports the critical role of the host immune response in oncogenesis. Adaptive immunity can shape a spontaneous anti‐tumor response while, in opposition, malignant cells can evade immune destruction through the promotion of an immunosuppressive tumor microenvironment (TME). Within EOC, the fraction and type of tumor‐invading lymphocytes (TILs) have been shown to influence disease prognosis and treatment response.^2^, ^3^, ^4^ In particular, strong data support the prognostic significance of cytotoxic CD8^+^ T cells, which directly induce tumor cell destruction.^5^, ^6^ Non‐cytotoxic CD4^+^ helper T cells may also contribute to a more favorable clinical outcome.^2^ Conversely, FoxP3^+^ regulator T cells (Tregs), through their immunomodulatory role, have been correlated with therapeutic resistance and worse survival outcomes in many solid tumors, although their role in ovarian cancer is still not completely defined.^5^, ^7^, ^8^, ^9^

Activin is a hormonal member of the TGF‐β superfamily named for its activating influence on the hypothalamic–pituitary‐gonadal axis^10^ and has been identified in a broad range of human tissue types and implicated in non‐reproductive activity, including in the maturation of both innate and adaptive immune cells as reviewed in.^11^ Activin has been shown to contribute to the differentiation and development of several immune cell subsets, including T cells and natural killer (NK) cells.^12^ Activin also participates in the recruitment of immune cells to sites of inflammation or infection, thereby modulating the effective immune response to pathogens or tumor cells.^13^, ^14^ Understanding the role of activin in immunity may provide valuable insights for the development of immunotherapeutic strategies in ovarian cancer.

Activin is a dimeric ligand composed of Inhibin‐βA (*INHBA*) or Inhibin‐βB subunits (*INHBB*) which bind activin type II receptors (*ACVR2A*, *ACVR2A*) as well as activin type I receptors (*ACVR1B* and *ACVR1C*) leading to phosphorylation of the SMAD2/3 transcription factors reviewed in [11, 12, 15]. In the context of cancer, knockdown of *INHBA* in mice leads to fatal adnexal masses.^16^ However, prior exploratory analyses that have examined *INHBA* gene expression profiles in different solid malignancies suggest that levels of *INHBA* expression may correlate with disease‐specific survival outcomes.^17^ Additional studies also implicate activin as a biomarker of malignancy, with potential diagnostic and prognostic implications.^18^, ^19^, ^20^, ^21^ Similarly, activin has demonstrated tumor promoting effects (survival, invasion and migration) in different cancer types.^22^, ^23^, ^24^ In EOC, activin‐A has been found to be elevated in both serum and ascites when compared to healthy controls, suggesting potential contextual effects of activins in cancer.^21^, ^25^ We thus sought to better characterize the activin expression profile within epithelial ovarian cancer with respect to tumor infiltrating immune cells and to assess for differences between primary and metastatic sites of disease. We found that activin expression within primary tumor and ascites burden is positively correlated with tumor immune infiltration and associated with poor disease prognosis. Our findings implicate the prognostic and therapeutic potential of activin‐A in epithelial ovarian carcinoma.

## 2 MATERIALS AND METHODS

### 2.1 Tissue specimen collection and processing

IRB approval was obtained prior to all human specimen collection, with studies conducted in accordance with the principles of the Belmont Report. Tissue from patients who had undergone surgical debulking for high grade serous EOC and patients who had undergone surgery for benign pathology were identified by the University of Alabama‐Birmingham Tissue Biorepository, with formalin‐fixed paraffin embedded tissue microarrays (TMA) generated at the tissue biorepository core. Patient demographics and clinical data were obtained by review of the electronic medical record. Overall survival (OS) was defined as the number of months from date of initial tissue diagnosis to date of death for deceased patients, or last clinic visit for patients alive with or without disease.

### 2.2 Bioinformatics

Publicly available RNA sequencing data of high grade serous ovarian carcinomas were accessed from GEO (#GSE143897).^26^ SRA meta‐data and accession list were downloaded onto the University of Alabama at Birmingham CHEAHA supercomputer using SRA‐Toolkit (version 2.9.6.1). The index for Salmon was built using GENCODE Release 19 (GRCh37.p13).^27^ Quantifications from Salmon were then analyzed in R (version 4.2.2).^28^ The quantifications were imported with tximport (version 1.26.1) using the same GENCODE reference, GenomicFeatures (version 1.50.4) (11), and TxDb.Hsapiens.UCSC.hg19.knownGene (version 3.2.2).^29^, ^30^, ^31^ Normalization and downstream analysis for gene quantification was performed with DESeq2 (version 1.38.3).^32^ Metadata are from the GSE143897 series matrix file.

TCGA‐OV data were extracted from the TCGA Research Network (https://www.cancer.gov/tcga) using the recount3 project within R (version 1.8.0).^33^, ^34^ We used the 430 samples present for differential gene expression. Metadata were downloaded from within the data matrix present in recount3, including FIGO stage. Differential expression analysis was performed utilizing with DESeq2.^32^ *INHBA* expression was regarded as the dividing point to stratify patients into high (70th percentile), intermediate (30th–70th percentile), or low (30th percentile) expression groups. Figures were made utilizing tibble and ggplot2. Code available at: https://github.com/page22emily/Activin.git.

### 2.3 Survival analysis

Survival data were generated from the online database KM Plotter, using the Affymetrix Probe ID *INHBA* (204926_at) and *INHBA* (2105511_a_atl). OS was assessed without threshold cutoff for follow‐up. Gene expression was split into high and low using the median expression, and log‐rank statistics were utilized to calculate both *p*‐value and hazard ratio (HR). The tool can be accessed at: https://kmplot.com/analysis/.^35^

### 2.4 Immunohistochemistry

Antibody optimization was performed with the support of the University of Alabama‐Birmingham Pathology Core Research Laboratory. Following deparaffinization of the tissue microarrays with sequential xylene, graduated ethanol and water washes, heat‐induced epitope retrieval was performed within sodium citrate buffer (pH 6.0) for anti‐CD8 (1:50 dilution, BioCare, #CRM311A), anti‐FoxP3 (1:100 dilution, BioCare, #ACI 3197A) and anti‐activin A (1:50 dilution, R&D Systems #AF338‐SP) antibodies. Endogenous peroxidases were blocked with 3% hydrogen peroxide followed by primary antibody incubation within a humidified chamber at 4°C overnight. Tissue microarrays were then treated with appropriate secondary antibody conjugated to horseradish peroxidase in blocking solution. HRP signal then detected with 3,3′‐diaminobenzidine (DAB; BioCare #BDB2004) substrate, washed then counterstained with Mayer's hematoxylin.

The stained TMA sections were digitally scanned using the Aperio CS digital slide scanner (Leica Biosystems Division of Leico Microsystems). Images were first accessed using Aperio ImageScope (version 12.3.4.5008), with each core then isolated and converted into tiff format for compatibility with ImageJ software. For quantification of tumor‐infiltrating immune cells, CD8 and FoxP3 levels within each core were manually counted using ImageJ software (NIH, version #1.53p), then normalized by nuclear count.^36^ For activin‐A levels, semi‐quantitative analysis was achieved using a 3‐tiered scoring system (low, medium, high). All quantitative and semi‐quantitative analysis was performed independently and blindly by a board‐certified pathologist and a second investigator, with discrepancies between the two investigators addressed.

### 2.5 ELISA

Single‐plex enzyme linked immunosorbent assay (ELISA) was performed using Activin‐A ELISA kit purchased from AnshLabs (AL‐110). ELISA was performed per the manufacturer's instruction to quantify activin A levels within ascites. Banked ascitic fluid was obtained from patients with high grade serous ovarian adenocarcinoma who had undergone therapeutic paracentesis. Ascitic fluid was obtained and stored after separation from the cell pellet. Patient fluid was obtained after informed consent subsequent to protocol approval by the University of Alabama‐Birmingham Institutional Review Board.

### 2.6 Statistical analysis

Statistical analysis was performed via GraphPad Prism version 9.5.1. RNA Sequencing data was analyzed separately, as described under bioinformatics. Data are reported as means, ± standard error of the mean (SEM) unless otherwise noted. Figure error bars reflect SEM. Multiple comparisons were evaluated using one‐way ANOVA, with Tukey post‐hoc analysis of a significant ANOVA group effect then performed as indicated. A *p* value of ≤0.05 was considered significant for all analysis. Survival curves were analyzed with log‐rank statistics.

## 3 RESULTS

### 3.1 Activin and activin receptor gene expression is elevated within malignant ovarian tissue

**FIGURE 1:**
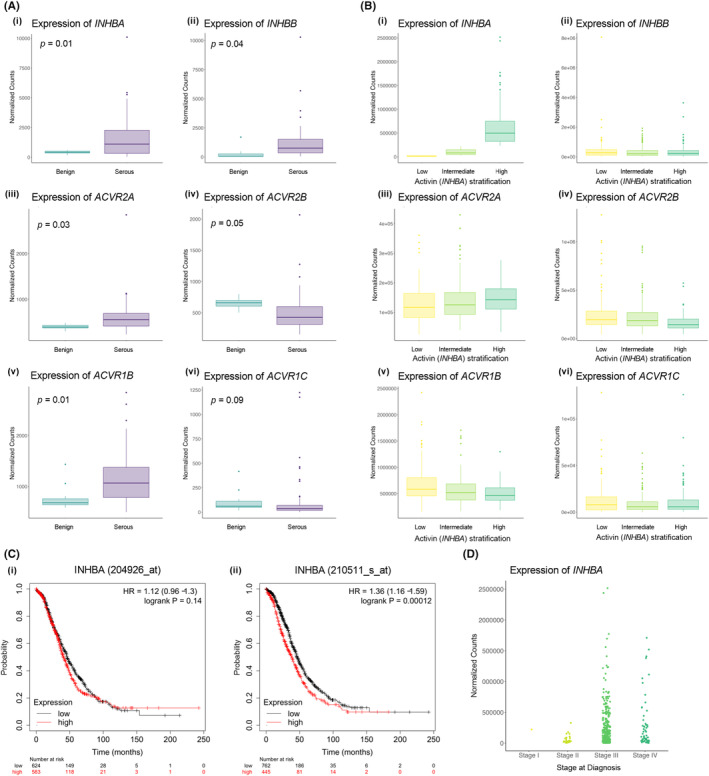
Serous ovarian carcinomas harbor transcriptional differences in activin and receptor gene expression when compared to benign ovarian tissues. (A) Analysis of indicated genes from GSE143897 containing 79 serous ovarian carcinoma patients and 11 benign tissues. (i–vi) Genes encoding for activin subunit *INHBA* and *INHBB* and activin type I and II receptors were found to be upregulated when compared to the benign cohort. (B) Analysis of indicated gene counts (*ACVR2A/2B* in iii–iv and *ACVR1B/1C* in v–vi) from TCGA‐OV dataset (*n* = 430) in samples stratified into low (< 30th percentile), intermediate (30th–70th percentile), and high (≥ 70th percentile) *INHBA* expression levels as shown in (i–ii). (C) Survival data were generated from KM Plotter,^32^ using the Affymetrix Probe ID *INHBA* (204926_at) and *INHBA (*2105511_a_atl). Gene expression was split into high and low *INHBA* using the median expression. High *INHBA* expression was associated with significantly worse overall survival in EOC patients. (D) Normalized counts of *INHBA* levels by stage in TCGA‐OV dataset (*n* = 430).

To evaluate gene level differences in components of the activin signaling pathway between benign and malignant ovarian tissue, we accessed publicly available, previously published RNA sequencing data (#GSE143897)^26^ (Figure 1A,B). This compilation contained transcriptional gene expression data from 79 patients with stage II–IV high grade serous EOC, 61 of whom were chemo‐naïve, 18 with prior platinum‐based chemotherapy exposure) and 11 benign ovarian tissues. Patient demographics and clinical characteristics of this cohort have been previously described but are consistent with clinical trends of the U.S. ovarian cancer patient population.^26^

Normalized counts of *INHBA* (422.6 ± 34.63, *p =* 0.02) and *INHBB* (1572 ± 187.8, *p* = 0.04), encoding the most prevalent subunits of the dimeric activin ligand, were assessed, and found to be elevated in EOC when compared to benign tissue (438.43 ± 37.36 and 262.7 ± 137.9, respectively) (Figure 1A). Differences in the gene expression of the activin type II receptors was also examined. While *ACVR2A* was found to be 1.5‐fold elevated in EOC compared to benign tissue (Figure 1A (iii)), *ACVR2B* expression was reduced by 1.3‐fold (Figure 1A (iv)). A similar pattern was found within the type I receptors, with *ACVR1B* 1.5 fold‐higher in malignant tissue (1152, 54.14) while *ACVRIC* level was not significantly different between cohorts (Figure 1A (v–vi)). Altogether, several components of the activin pathway were found to be elevated at the gene level within malignant serous epithelial ovarian tissue (EOC) when compared to the benign cohort (Figure 1A,B, Table S1).

To allow for external validation of our findings within a larger more heterogenous sample population, and to assess whether *INHBA* expression was correlated with receptor expression, we turned to the TCGA‐OV dataset derived from the TCGA PanCancer Atlas (Figure 1B). Four hundred thirty patients with serous ovarian carcinoma were identified. The distribution of *INHBA* expression by advancing stage of disease is demonstrated in Figure 1D. *INHBA* expression values were used as the dividing point to stratify patients into high (70th percentile), intermediate (30th–70th percentile), or low (30th percentile) expression groups. *ACVR2A* expression trended toward a positive correlation with increasing *INHBA*, while the inverse was noted with *ACVR1B*, however this did not reach significance. No differences in expression profiles were noted with *ACVR2B* or *ACVR1C* when stratified by *INHBA* expression (Figure 1B). Furthermore, high *INHBA* expression was found to be associated with significantly worse overall survival in patients with ovarian cancer, as demonstrated by Kaplan–Meier curves presented in (Figure 1C (i–ii)) and analyzed using Kaplan–Meier Plotter, an open‐access meta‐analysis tool which compiles gene expression data from TCGA, GEO, and EGA databases.^37^ “Jet set” probes, *INHBA* (204926_at) and *INHBA* (210511_s_at) were used to generate KM Curves of serous ovarian carcinoma, stratified by high and low *INHBA* expression. No significant survival differences was noted when stratified by *INHBB* expression (HR 1.1, *p =* 0.21). For *INHBA* two probe ID's were identified. While no statistically significant difference in survival was reached with one of the ID's for *INHBA* (Figure 1C (i), 204926_at), high *INHBA* using 210511_s_at (Figure 1C (ii)), defined as transcriptional gene expression above the median expression level, was associated with a poor prognosis (HR 1.36, *p* < 0.001). Median OS of the high expression cohort was 40.54 months, compared to 45.77 within the low *INHBA* cohort.

### 3.2 A lymphocytic gene profile was positively correlated with activin A expression

**FIGURE 2:**
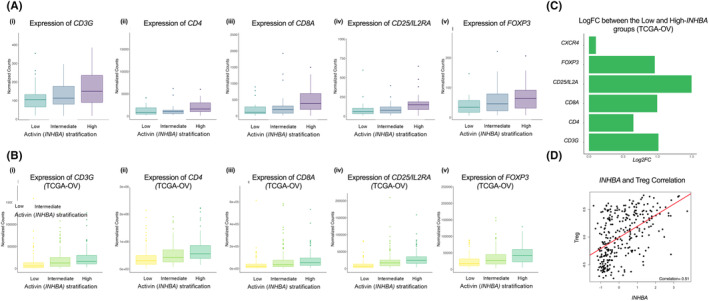
A lymphocytic gene profile was positively correlated with *INHBA* (Inhibin‐βA) expression. Normalized counts of indicated markers of lymphocyte genes in the samples stratified into *INHBA* low (<30th percentile), intermediate (30th–70th percentile), and high‐expression groups (≥70th percentile) from the GSE143897 cohort (A) and the TCGA‐OV cohort (B). (C) Log fold change increases in the indicated lymphocytic gene profile when comparing Low and high *INHBA* patient cohort from TCGA. (D) Pearson Correlation analysis (*r* = 0.51) between the *z* score of *INHBA* in TCGA‐OV patients and Treg signatures from.^40^

Prior studies have suggested a potential association between activin and immunity, but the influence of activin within the EOC immune TME is not well characterized.^12^ To test the relationship between *INHBA* expression and immune signatures, we analyzed a panel of genes encoding for specific tumor‐infiltrating lymphocyte (TIL) cell types (Figure 2A). When GSE143897 cohort was stratified by *INHBA* expression, *INHBA* level was positively correlated with *CD3G, CD4, CD8A, FOXP3* and *CD25* (Figure 2A (i–v), although statistical significance was only reached for *CD8A* (*p* = 0.03) and *FOXP3* (*p* = 0.02). Similarly, TCGA analysis demonstrated a positive association between increasing *INHBA* expression and lymphocytic gene profile, with *CXCR4, FOXP3, CD25/IL2A, CD8, CD4* and *CD3G* expression being higher in high‐*INHBA* cohort as compared to the low‐*INHBA* cohort (Figure 2B,C). To further assess this correlation between *INHBA* and genes associated with T cell function, we tested *INHBA* z score levels as extracted from cBioportal^38^, ^39^ for ovarian tumors against tumor immunophenotypes as defined from 9174 tumors of 29 solid cancers.^40^ We find that when comparing *INHBA* levels against the 16 immune population signatures, the most significant Pearson's correlation was seen with a Treg signature (Figure 2D). Hence together these data suggest that across patient cohorts, *INHBA* is correlated with lymphocytic gene expression, favoring a more immunomodulatory profile.

### 3.3 Tissue activin levels correlate with lymphocytic infiltration of the tumor microenvironment

**FIGURE 3:**
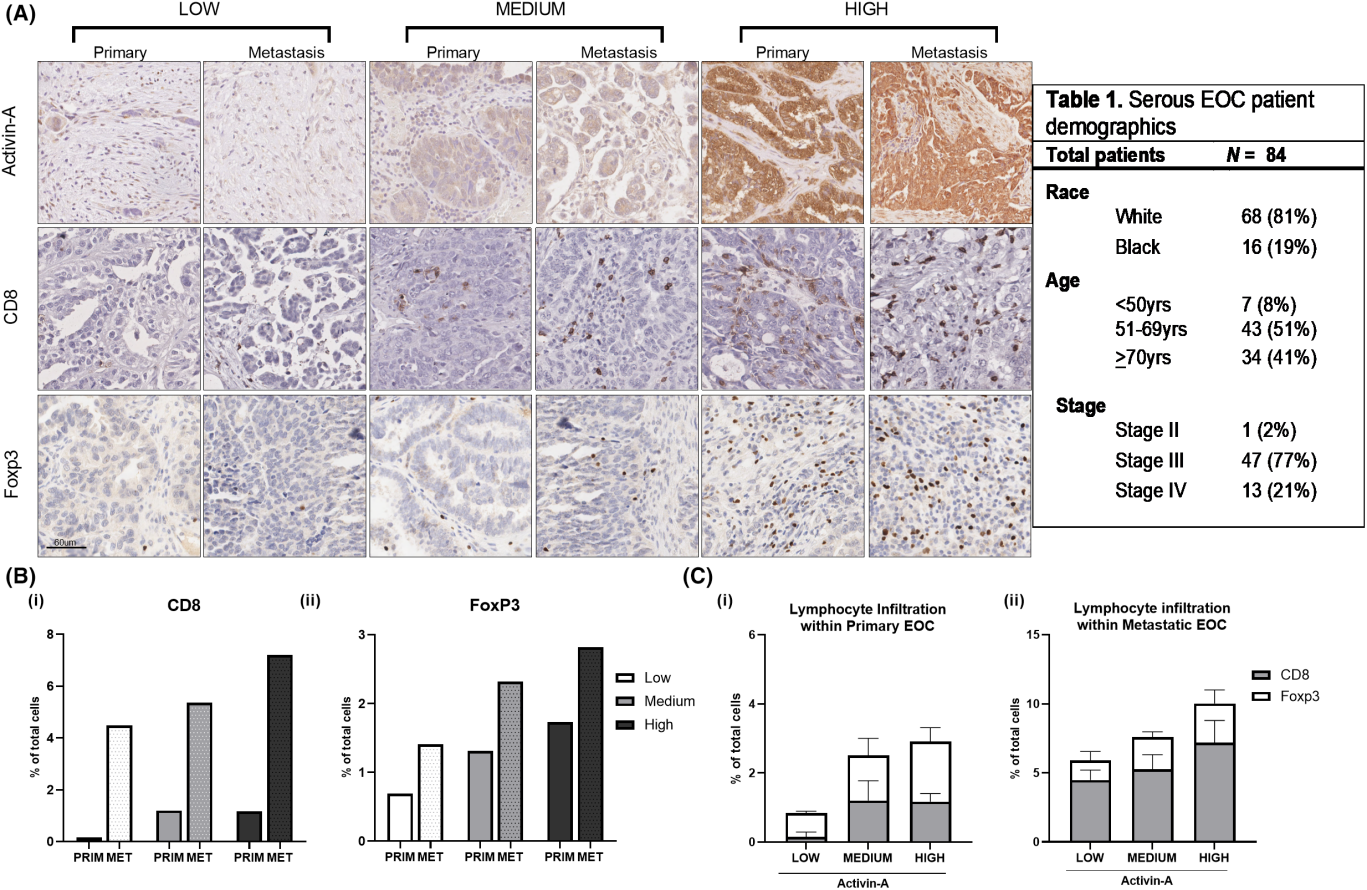
Within ovarian carcinoma tissue, CD8 and Foxp3 levels are positively correlated with activin‐A staining. (A) Representative images of indicated proteins (Foxp3, CD8^+^ and activin A) from immunohistochemical staining of ovarian cancer TMAs composed of either primary or metastatic serous EOC from 83 chemo‐naïve patients (44 primary sites of disease, 39 metastatic). Each image was taken from the center of tissue core at 40× magnification. (B–C) Quantitation of CD8 and Foxp3 positive cells in the entire core normalized to the total number of nuclei, stratified by low, medium and high activin‐A levels in the cores. Higher total immune infiltration was seen in those with high activin‐A. When stratified by activin‐A, both CD8 and FoxP3 expression within primary tumors positively correlated with activin‐A, with CD8^+^ level 7‐fold higher in the high activin‐A cohort than the low, while FoxP3 was 1.6 times greater.

The majority of EOC patients have metastatic disease at time of diagnosis.^41^ Immune infiltration differences between primary and metastatic sites has been reported, with differences in the immune cell infiltrate positively associated with response to neoadjuvant chemotherapy.^42^ Given the correlation between *INHBA* gene expression and TIL (Figure 2), we sought to assess differences in activin‐A protein levels and immune infiltration in primary and metastatic sites of disease. Immunohistochemical analysis of an ovarian cancer TMA composed of primary and metastatic serous EOC from 83 chemo‐naïve patients (44 primary sites of disease, 39 metastatic) from University of Alabama were analyzed. Patient demographics are summarized in Figure 3. TMAs were stained for activin‐A, as well as CD8 and FoxP3, to assess a cytotoxic and immunosuppressive phenotype, respectively (Figure 3A). Both immune cell phenotypes were detected at a higher fraction within the intrastromal region of the tissue cores than in the intratumoral region, consistent with what has been previously described.^43^ CD8 and FoxP3 expression was quantitatively analyzed via manual counting of the number of positive cells, which was then normalized by the number of nuclei within the core to determine the percentage of core‐positive cells. Both CD8^+^ (5.77%, ± 0.86) and FoxP3^+^ (2.38%, + 0.36) immune‐infiltration fraction was noted to be higher within the metastatic cores than within primary cores (0.97%, ± 0.23 and 1.40% ± 0.24, respectively) (Figure 3B).

Activin‐A was detectable in both primary and metastatic cores, and was overall significantly higher in the metastatic cohort (Figure 3B). No significant difference in activin‐A were noted when tissue was stratified by BMI or race. Given the nature of the tumor and stromal staining pattern, staining was qualitatively grouped into activin‐A–low, activin‐A–medium, and activin‐A–high expression by a board certified pathologist. When stratified by activin‐A level, both CD8 and FoxP3 positivity within primary tumors positively correlated with activin‐A, with CD8^+^ level 7‐fold higher in the activin‐A–high cohort than the activin‐A–low, while FoxP3 was 1.6 times greater (Figure 3C (i–ii)). Total immune infiltration mirrored this pattern, with higher total immune infiltration measured in the activin‐A–high cohort.

The association between total immune infiltration and activin‐A persisted when primary and metastatic sites were analyzed individually, although this did not reach significance (*p* = 0.717), nor was a significant difference in CD8^+^ to FoxP3^+^ ratio appreciated (*p* = 0.828). However, a significant difference was noted in CD8^+^ cells between primary and metastatic cores when stratified by activin‐A with the difference in the relative fraction of CD8^+^ cells within metastatic cores as compared to primary greatest in the high activin‐A cohort (Figure 3B). Altogether, this suggests a potential difference in expression pattern at primary and metastatic sites both with respect to activin A and its correlation with CD8 and FoxP3.

### 3.4 Activin‐A is elevated in EOC patient ascites

**FIGURE 4:**
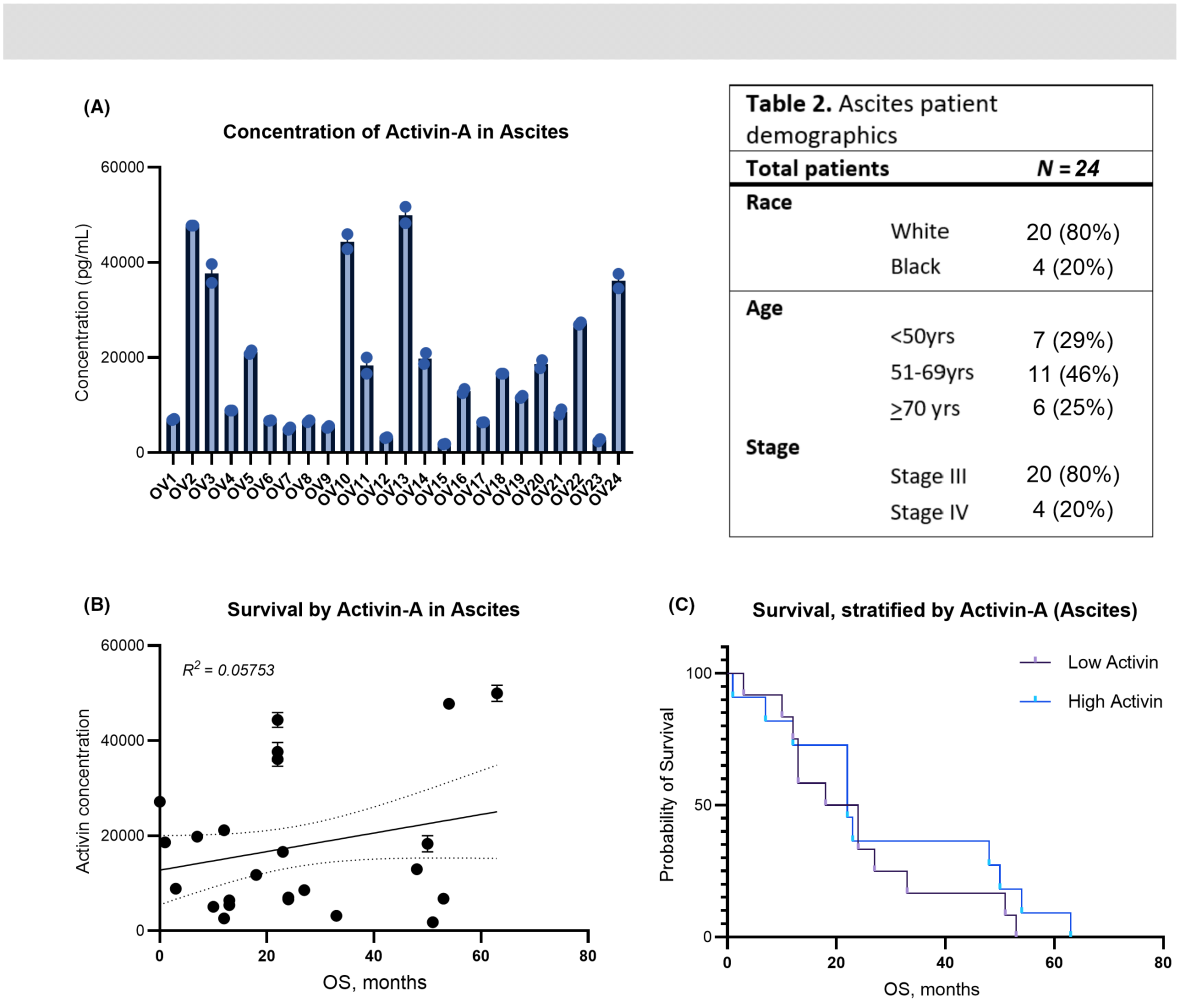
Activin‐A is elevated in patient ascites but is not a robust predictive marker of disease survival. (A) Concentration (pg/mL) of activin A in the ascites of 24 patients with advanced high grade serous EOC. Median concentration was 12,356 pg/mL. (B) ROC curve analysis to assess correlation between activin‐A level and overall survival. (C) Kaplan–Meier curve of patients stratified by median activin‐A concentration (activin‐low and activin‐high).

Prior reports on activin‐A have identified measurable levels within both serum and ascites of ovarian cancer patients, suggesting its utility as a biomarker for malignancy.^21^, ^25^ We also performed an ELISA to detect activin‐A within the ascites of 24 patients from a single institution, with advanced high grade serous EOC (Figure 4A). Median concentration was 12,356 pg/mL, similar to what was previously reported in malignant peritoneal fluid, and approximately 12‐fold greater than that found in historical benign controls in circulation.^21^ Patients were then stratified by median activin‐A in ascites. Given the observed correlation between solid tumor *INHBA* expression and *s*urvival outcomes (Figure 1C), we expected to observe a similar association within the ascites samples. However, using Cox log‐rank hazards regression to analyze the influence of activin‐A levels in ascites on patient OS, we found that activin‐A level in ascites did not correlate strongly with survival outcomes (HR 0.95, 95% confidence interval 0.42–2.16) (Figure 4B,C). While prior studies have suggested a negative correlation between activin A levels and disease prognosis, these findings suggest that the prognostic utility of high activin expression may be site and tissue specific.^21^, ^25^

## 4 DISCUSSION

This study sought to evaluate the expression profile of activin and a subset of its receptors within epithelial ovarian cancer. Our findings lend support to activin as a player in the ovarian cancer tumor microenvironment, shedding light on a potential role in the mediation of the lymphocytic immune profile within primary and metastatic sites of disease. Together, both RNA levels of *INHBA* and protein analysis implicate activin‐A as a potential prognostic marker of disease survival.

While much work has been done elucidating the role of TGF‐β on cancer cell proliferation and metastasis, less is known about the other members of the TGF‐β superfamily, including activins. As presented here, not only was *INHBA* expression found to be elevated within EOC when compared to benign controls, but key transcriptional differences in the receptor components were identified, suggesting discrete signaling cascades between malignant and benign ovarian cohorts. The relative upregulation within the malignant cohort of *ACVR1B*, encoding activin type I receptor ALK4, and downregulation of *ACVR1C*, encoding activin type I receptor ALK7, is consistent with prior reports of preferential binding of activin to ALK4‐containing receptor complexes.^44^ ALK4‐mediated signaling has been implicated in promoting a tumorigenic phenotype through the promotion of cancer cell proliferation and invasion.^45^, ^46^ In contrast, ALK7 has been associated with decreased tumor proliferation in breast and prostate cancers.^47^, ^48^ Together, this suggests that the pro‐oncogenic activin phenotype may be the result of its preferential ALK4 signaling, but further studies are required to explain what tips the balance toward either cascade.

Our analysis of a publicly available sequencing dataset is in concordance with prior studies, which have consistently supported the prognostic utility of both serum and tissue activin expression across multiple cancer types, including lung, breast, colorectal, pancreatic and melanoma.^17^, ^21^, ^25^ While one clear limitation of the work presented here is the sample size of the cohort explored, our validation within the larger TCGA database supports our findings. The differences in activin expression across primary and metastatic sites of disease, as well as within ascites itself, may be representative of tumor heterogeneity arising from different oncogenic drivers. The overexpression of activin appreciated on IHC staining of metastatic tissues, points to activin playing a direct or indirect role in the promotion of metastatic cell proliferation and survival.

It is particularly interesting that gene expression correlates with worse disease prognosis and activin protein levels correlate with immune infiltration differences. The immunogenicity of ovarian cancer has been characterized, with the magnitude of T cell infiltration within disease burden correlating with longer survival.^3^, ^43^ However, despite the intratumoral immune presence, ovarian cancer response to immunotherapy has been disappointing. This has been shown to be due at least in part to localized immunomodulation, with FoxP3^+^ regulatory T cells as well as other immune checkpoint regulators, such as induced programmed cell death (PD‐1) that together may limit antitumor immune response.^49^, ^50^, ^51^ Activin has been implicated in the promotion of a CD4^+^/Foxp3^+^ phenotype in a dose‐dependent manner in both malignant (breast cancer) and non‐cancerous disease models.^13^, ^14^, ^52^ Furthermore, the suppression of activin signaling restores CD8^+^ and CD4^+^ T cell infiltration and correlates with slowed tumor growth in breast cancer.^13^ It is possible that similar outcomes may occur in ovarian cancer and need to be examined.

However, whether this increase is due to direct activin‐mediated immune recruitment, T cell or an indirect effect on angiogenesis or other stromal cells is unclear as activin is also present in non‐ tumor cells in EOC. VEGF is a pro‐angiogenic factor highly expressed in ovarian cancer, promoting neovascularization and immunomodulation by inhibiting T cell migration and promoting an immunomodulatory T cell phenotype.^53^, ^54^ The role of activin in angiogenesis is not well understood, but it appears to hinder vascular endothelial cell growth in both benign and cancerous models.^55^, ^56^ Since FDA approval, anti‐VEGF therapy is commonly utilized in ovarian cancer with its therapeutic benefit thought to arise not only from disrupted tumor angiogenesis but also disrupted immune evasion. Attempts to further mitigate this immunomodulation have achieved some success, with a recent phase II trial combining immune checkpoint inhibitor cyclophosphamide with bevacizumab demonstrating a greater response rate than historically reported with bevacizumab alone.^57^ Our findings suggest activin may be another viable addition, alone or in combination with bevacizumab, to an immunotherapy regimen that could allow for the circumvention of immunotherapy resistance. Further investigation into the adjuvant use of activin in targeted therapy is warranted, to better determine the clinical relevance of the immunomodulatory findings presented here.

## 5 CONCLUSIONS

*INHBA* expression is elevated within EOC, with gene expression correlating with worse survival and activin protein levels correlating with specific immune infiltration that may lead to suppressing anti‐tumor immunity in EOC. The positive association between activin A and lymphocytic immune infiltration lends support to the role of activin as a mediator of the EOC immune environment. Together, our findings implicate this growth factor as a potential therapeutic target that warrants further investigation.

## AUTHOR CONTRIBUTIONS

**Elizabeth T. Evans:** Conceptualization (equal); data curation (equal); formal analysis (equal); funding acquisition (equal); investigation (lead); methodology (lead); project administration (lead); visualization (equal); writing – original draft (lead); writing – review and editing (equal). **Emily Page:** Formal analysis (equal); investigation (equal); visualization (equal); writing – review and editing (equal). **Alex Seok Choi:** Investigation (supporting); methodology (supporting); validation (equal); writing – review and editing (supporting). **Zainab Shonibare:** Investigation (equal); visualization (equal). **Andrea G. Kahn:** Formal analysis (supporting); investigation (supporting). **Rebecca C. Arend:** Conceptualization (equal); writing – review and editing (equal). **Karthikeyan Mythreye:** Conceptualization (lead); formal analysis (lead); funding acquisition (lead); supervision (equal); visualization (equal); writing – review and editing (equal).

## FUNDING INFORMATION

Funding was provided by NCI T32CA229102 to Elizabeth T. Evans and NCI R01CA219495 to Karthikeyan Mythreye.

## CONFLICT OF INTEREST STATEMENT

RCA has performed a consultatory or advisory role at Merck, Seagen, Sutro, GSK, VBL therapeutics, and Kiyatec, and received research support from Immunogen, GSK, GOG Foundation, Champions Oncology, Exelixis, and Merck. All other authors have no conflicts of interest.

## Supporting information


Figure S1.

**Table S1:** Activin and activin receptor gene expression in malignant and benign ovarian tissue.

## Data Availability

The data that support the findings of this study are all in the manuscript and also available upon request from the corresponding author.
