# Supplementary material for: Activin levels correlate with lymphocytic infiltration in epithelial ovarian cancer
Source: Cancer Med. 2024 Sep 9;13(17):e7368. doi: 10.1002/cam4.7368 (PMC11381957; doi:10.1002/cam4.7368)
Supplement: Supplementary file 1 — Figure S1. [file CAM4-13-e7368-s001.docx]

**Supplemental Figure 1: Representative images of immunohistochemistry (IHC) performed on tumor microarrays (TMAs) composed of primary or metastatic human serous ovarian cancer and normal tissue controls.** TMA were stained with anti-activin (A), anti-CD8 (B) and anti-FoxP3 (C) antibodies before being scored. Black boxes represent the representative low-scoring images presented in Figure 2, while green represent the intermediate-scoring and red high-scoring.

**A. Anti-Activin staining**

| **(i)** Primary-EOC TMA | (**ii)** Metastatic-EOC TMA |
| --- | --- |
| 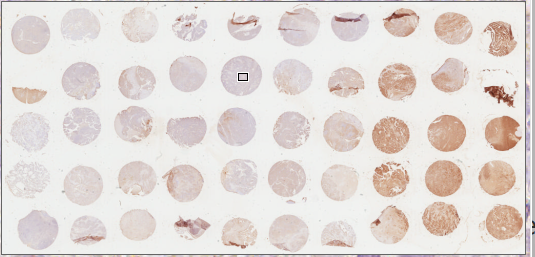 | 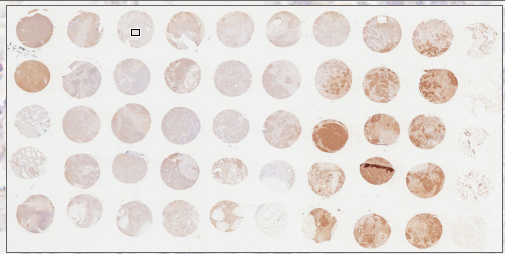 |

**B.** **Anti-CD8 Staining**

| **(i)** Primary-EOC TMA | **(ii)** Metastatic-EOC TMA |
| --- | --- |
| 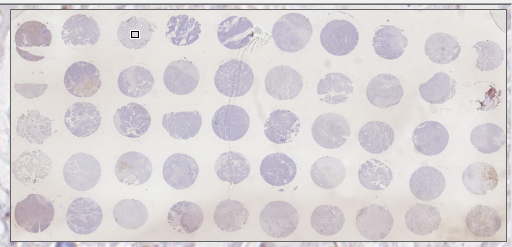 | 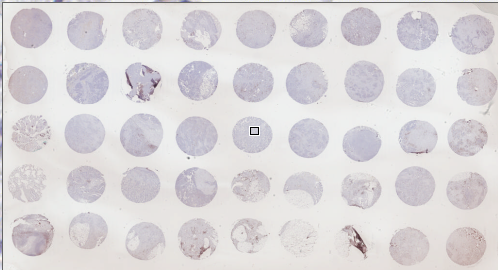 |

**C. Anti-FoxP3 Staining**

| **(i)** Primary-EOC TMA | **(iI)** Metastatic-EOC TMA |
| --- | --- |
| 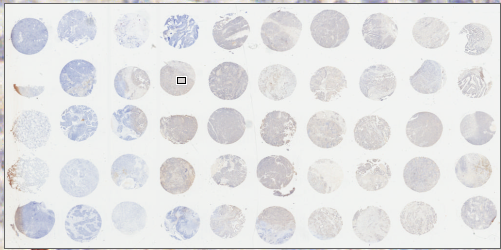 | 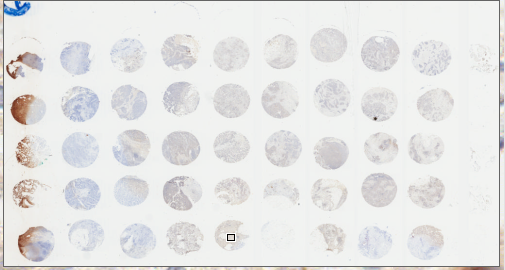 |
