# Supplementary material for: Activin levels correlate with lymphocytic infiltration in epithelial ovarian cancer
Source: Cancer Med. 2024 Sep 9;13(17):e7368. doi: 10.1002/cam4.7368 (PMC11381957; doi:10.1002/cam4.7368)
Supplement: Supplementary file 2 — Table S1: Activin and activin receptor gene expression in malignant and benign ovarian tissue. [file CAM4-13-e7368-s002.docx]

**Supplementary Table 1:**

|  | Benign | Serous EOC | Significance |
| --- | --- | --- | --- |
| *INHBA* | 422.6 + SEM 34.63 | 1572 + SEM 187.8 | *p* = 0.02 |
| *INHBB* | 272.7 + SEM 137.9 | 1193 + SEM 160.5 | *p* = 0.04 |
| *ACVR2A* | 398.7 + SEM 14.83 | 603 + SEM 37.36 | *p* = 0.03 |
| *ACVR2B* | 654.3 + SEM 23.5 | 488.5 + SEM 32.17 | *p* = 0.05 |
| *ACVR1B* | 772.7 + SEM 70.41 | 1152 + SEM 54.14 | *p* = 0.01 |
| *ACVR1C* | 108.3 + SEM 32.54 | 96.98 + SEM 23.20 | *p* = 0.09 |
